# Case report: Filariasis presenting as an intra-abdominal cyst

**DOI:** 10.4103/0971-3026.76048

**Published:** 2011

**Authors:** Abhay K Kapoor, Sunil Kumar Puri, Ankur Arora, Lalendra Upreti, Amrender S Puri

**Affiliations:** Department of Radiology, GB Pant Hospital, New Delhi, India; 1 Department of Gastroenterology, GB Pant Hospital, New Delhi, India

**Keywords:** Abdominal cyst, filaria, lymphadenovarix

## Abstract

Filariasis is an endemic infection seen in the tropical and subtropical regions of the world, presenting with lymphatic dysfunction in the form of lymphocele, hydrocele, chyluria, or groin lymphadenovarix. We report a rare presentation of filariasis as an intra-abdominal cystic mass.

## Introduction

Filarial infections are common in most tropical and subtropical regions of the world. Lymphatic filariasis is caused by *Wuchereria bancrofti, Brugia malayi*, or *Brugia timori*. The threadlike worm resides in the lymphatic channels or lymph nodes. The clinical manifestations are directly related to the occlusion of the lymphatic channels, thereby causing lymphangiectasia.[1] The most common presentations of lymphatic filariasis are subclinical microfilaremia, hydrocele, acute adenolymphangitis, and chronic lymphatic disease.[2] We report a rare presentation of filariasis as a retroperitoneal cystic lesion, the diagnosis of which was confirmed by the demonstration of live microfilariae in the cyst fluid.

## Case Report

A 42-year-old male patient was referred for radiological evaluation of a gradually enlarging lump, extending from the right hypochondrium to the infraumbilical region for the last 6 months. He also had occasional low-grade fever.

**Figure 1:**
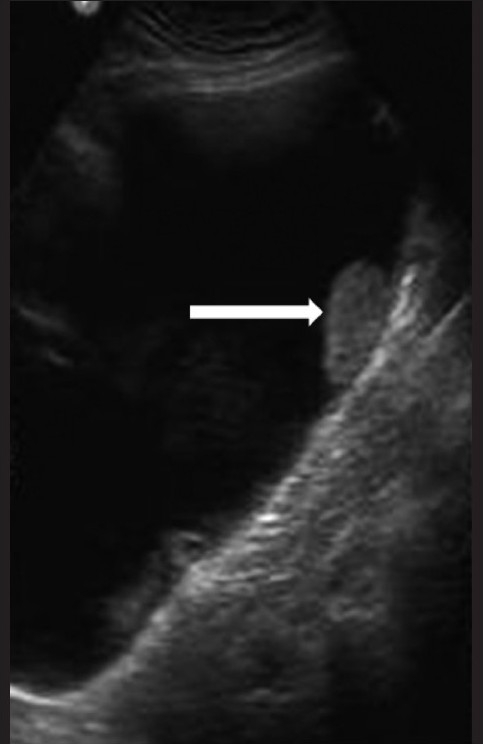
Transverse USG image of the right lumbar region of the abdomen shows a large intra-abdominal cystic lesion with a small solid component (arrow) and debris

**Figure 2 (A,B):**
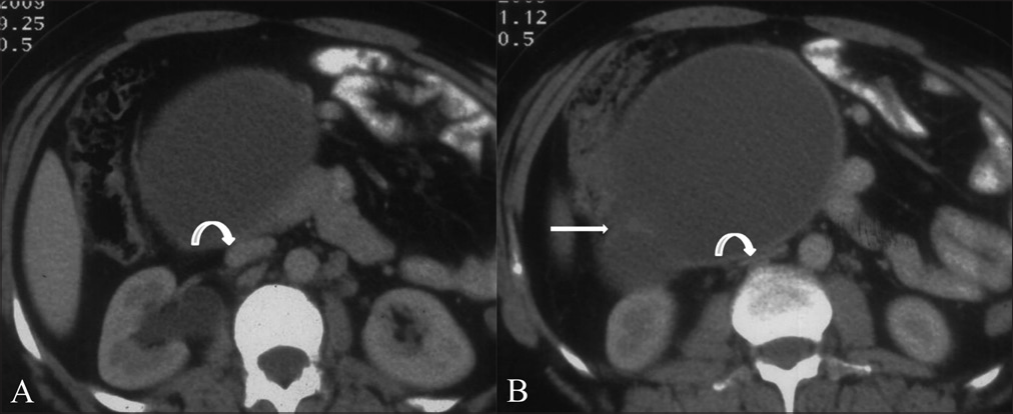
Contrast-enhanced CT scan of the abdomen at the level of inferior pole of the right kidney shows a cystic lesion with internal septae (arrow) anterior to the inferior vena cava (IVC). The IVC is compressed (curved arrow)

**Figure 3:**
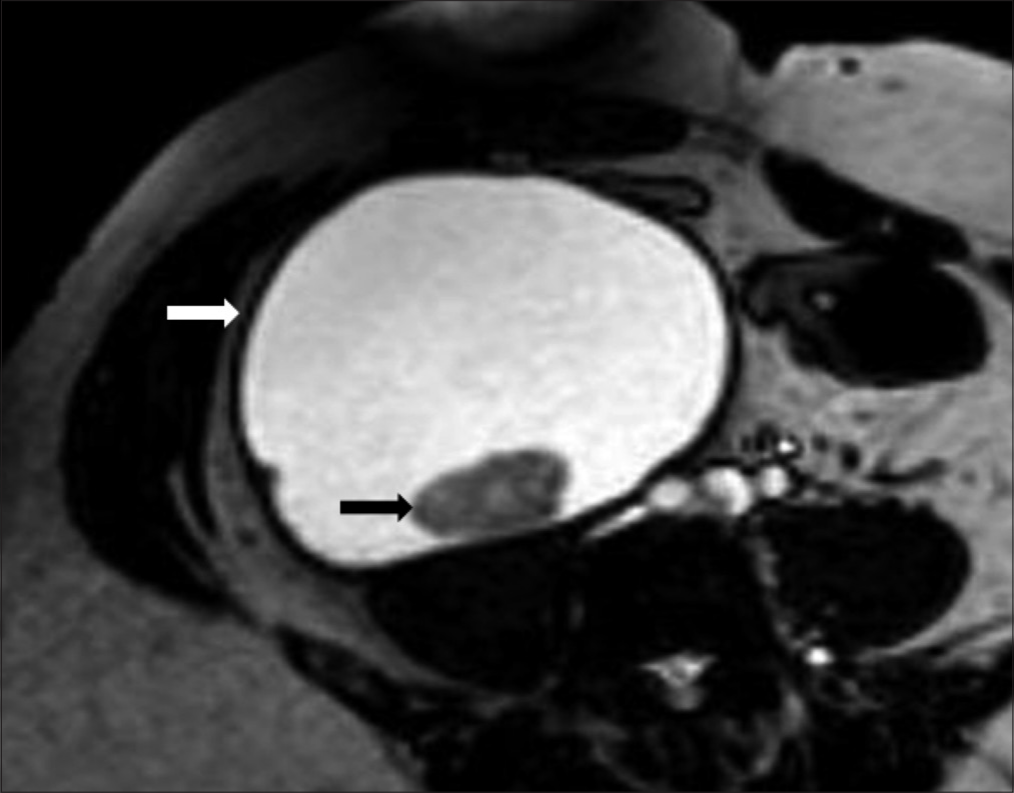
Axial, steady-state, free-precession MRI image shows a hyperintense lesion (white arrow) with a hypointense area within (black arrow). The lesion is predominantly hypointense on T1W and hyperintense on T2W images, indicating a cystic lesion containing debris

**Figure 4:**
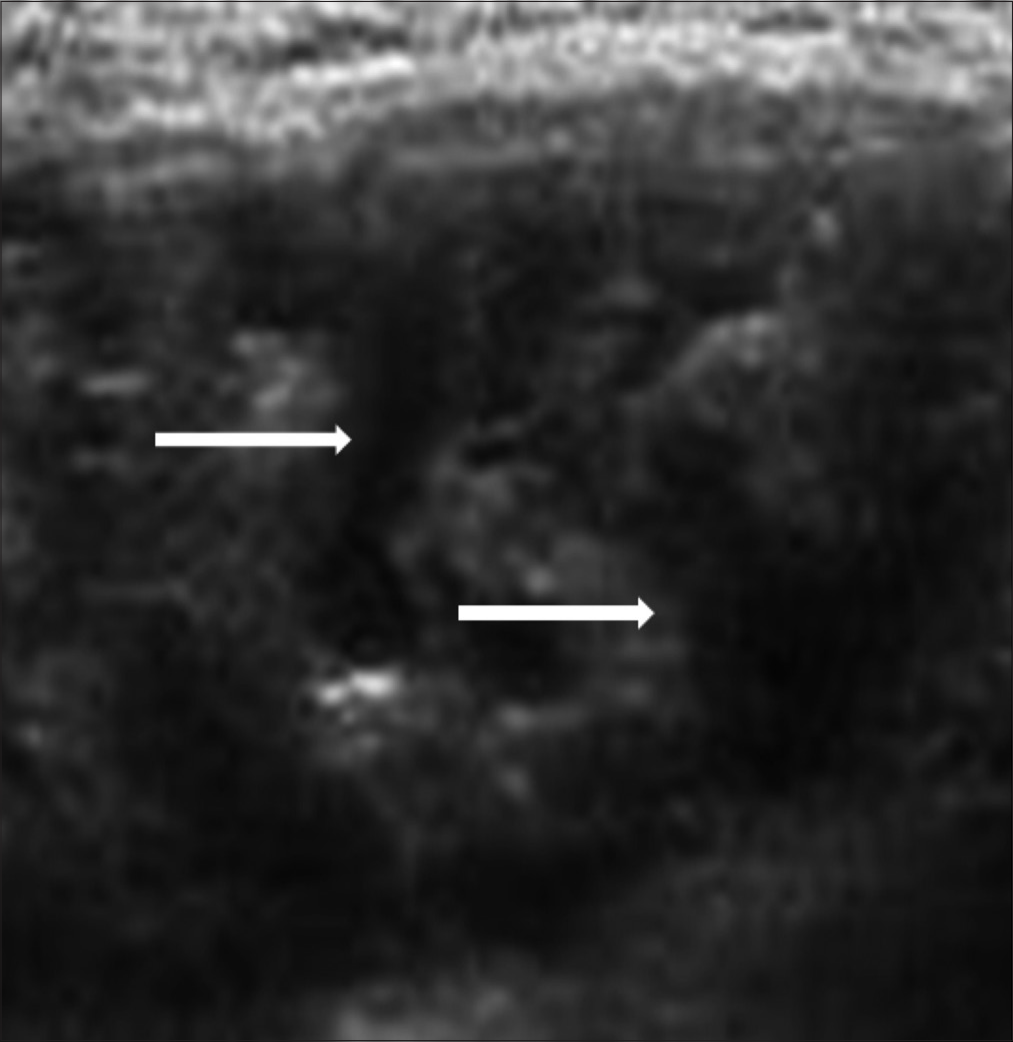
Transverse USG image of the scrotum shows diffuse lymphangiectasia (arrows) in the right inguinoscrotal region

**Figure 5:**
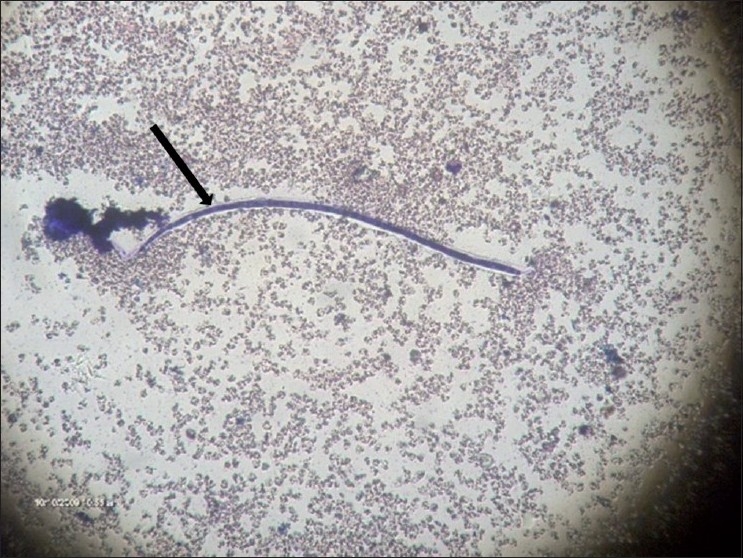
Magnified image of a smear of the aspirated cyst fluid shows microfilariae of *Wuchereria bancrofti* (arrow)

Ultrasonography (USG) revealed a cystic lesion extending from the subhepatic region to the pelvis. It showed internal septations, debris, and few small solid areas [Figure 1]. The patient underwent contrast-enhanced CT scan for further characterization. The cystic lesion was seen to lie in the retroperitoneum and cause compression of the inferior vena cava. No obvious enhancing solid component was seen [Figures 2A, B]. On MRI, the lesion appeared hypointense on T1W images and predominantly hyperintense on T2W and SSFP images, with hypointense debris within [Figure 3]. No blood products were detected. During the course of the evaluation, a history of a coexisting mild scrotal swelling was obtained. On high-resolution USG of the scrotum, a small hydrocele was seen on both sides with a thickened spermatic cord [Figure 4]. This additional finding suggested the possibility of a cyst of lymphatic origin. A fine-needle aspiration cytology (FNAC) was obtained. Chocolate-colored fluid was aspirated, which revealed live microfilariae of *Wuchereria bancrofti* on microscopic examination [Figure 5]. The patient was administered diethylcarbamazine as a provocative maneuver. Subsequent peripheral blood smear demonstrated motile microfilariae, confirming our diagnosis. The patient was put on diethylcarbamazine therapy for 4 weeks. Follow-up CT scan after 6 weeks did not show any significant change in the size of the lesion. At this point, surgical intervention was considered and the patient underwent laparotomy. On surgery, a well-defined retroperitoneal cyst was removed after careful dissection to release adhesions. Histopathology revealed the presence of microfilariae in the cyst. The postoperative period was uneventful, and the patient was discharged after 2 weeks.

## Discussion

Clinical examination alone is seldom helpful in the evaluation of an abdominal mass, apart from its ability to localize the lesion in some cases. Imaging in conjunction with clinical evaluation remains essential for more accurate localization, characterization, and for narrowing the differential diagnosis.

USG and CT scan help to differentiate between cystic and solid masses. The presence of a cystic retroperitoneal mass may be due to many entities including benign lesions such as lymphocele, urinoma, mesothelial cyst, lymhangioma, traumatic cyst, and parasitic cyst as well as malignant lesions such as mucinous cystadenocarcinoma, pseudomyxoma retroperitonei, and parachordoma. In our patient, we considered the possibilities of lymphangioma, traumatic cyst, or mesothelial cyst.

Despite extensive imaging evaluation, the diagnosis very often is only obtained on some form of histopathology. In our case as well, though the presence of an associated scrotal swelling suggested the possibility of a cyst of filarial origin, the diagnosis could be confirmed only after an FNAC.

Filarial retroperitoneal cysts have a reported incidence of 1 in 105 000 hospitalized patients.[3,4] There are only few, isolated case reports in the English literature,[4–7] all from the Indian subcontinent. The retroperitoneum is a rare location for a cyst, even in areas where filarial infection is endemic. The exact pathogenesis of the lesion remains speculative. Obstructed lymphatic vessels, rupture of lymphatics causing extravasation of chyle, and the presence of ectopic lymphatic tissue have been propounded as the possible etiologies.[6] Diethylcarbamazine is the drug of choice for the medical treatment of filariasis. Small retroperitoneal lesions may resolve with antifilarial therapy,[7] but most cases require surgical removal because of their large size, as in our case.
